# Changes in Balance Strategy and Neuromuscular Control during a Fatiguing Balance Task—A Study in Perturbed Unilateral Stance

**DOI:** 10.3389/fnhum.2016.00289

**Published:** 2016-06-14

**Authors:** Ramona Ritzmann, Kathrin Freyler, Amelie Werkhausen, Albert Gollhofer

**Affiliations:** ^1^Department of Sports and Sports Science, University of FreiburgFreiburg, Germany; ^2^Department of Physical Performance, Norwegian School of Sport SciencesOslo, Norway

**Keywords:** postural control, perturbation, electromyography, center of pressure, co-contraction, H-reflex, plasticity, sensorimotor control

## Abstract

Fatigue impairs sensorimotor performance, reduces spinal reflexes and affects the interaction of antagonistic muscles in complex motor tasks. Although there is literature dealing with the interference of fatigue and postural control, the interpretation is confounded by the variety of paradigms used to study it. This study aimed to evaluate the effects of postural fatigue on balance control and strategy, as well as on neuromuscular modulation, in response to postural perturbation (PERT) during a fatiguing balance task. A fatigue protocol consisting of continuous exposure to perturbations until exhaustion was executed in 24 subjects. Number of failed attempts, paths of center of pressure displacement (COP), ankle, knee, and hip joint kinematics, electromyographic activity of the soleus (SOL), tibialis anterior (TA), rectus femoris (RF), vastus lateralis (VL), biceps femoris (BF), and gluteus maximus muscles (GM) and spinal excitability of SOL at the peak of the short-latency responses (SLR) were recorded after posterior PERT. The co-contraction index (CCI) was calculated for TA_SOL, VL_BF and RF_GM. (1) The number of failed attempts significantly increased while COP amplitude and velocity, as well as angular excursion at the ankle, knee and hip joints, decreased with fatigue (*P* < 0.05). (2) Concomitantly, CCI of SOL_TA, VL_BF and RF_GM increased and spinal excitability in SOL declined. (3) Adaptations progressively augmented with progressing exhaustion and occurred in the distal prior to proximal segment. Distinctly deteriorated balance ability was accompanied by a modified neuromuscular control—the increase in co-contraction reflected by simultaneously activated antagonists is accompanied by smaller knee and hip joint excursions, indicating an elevated level of articular stiffness. These changes may be associated with an exaggerated postural rigidity and could have caused the delayed and reduced postural reactions that are reflected in the changes in COP displacement when compensating for sudden PERT. The reduction in spinal excitability may either be caused by fatigue itself or by an increase in reciprocal inhibition due to augmented TA activity.

## Introduction

Body equilibrium is maintained by joint torques, which are controlled by the central nervous system (CNS). Postural adjustment in response to perturbation requires the detection of body segment movements via visual, vestibular and proprioceptive sensory afferents, integration of that information into the CNS and the execution of an appropriate motor response (Dichgans et al., 1976; Nashner, 1976; Nashner and Berthoz, 1978; Dietz, 1992). Fatigue—by definition, an exercise-induced decline in muscle force—affects this sensorimotor coupling and, thus, considerably threatens body equilibrium (Vuillerme et al., 2002; Gribble and Hertel, 2004).

Fatigue-induced deterioration in balance control is associated with an increased sway path (Nardone et al., 1997; Kelly et al., 2005; Berger et al., 2010) and velocity (Kelly et al., 2005; Bisson et al., 2011), augmented variability of the postural response (Bisson et al., 2011), changes in neuromuscular activation reflected by modulated spinal reflexes (Herrmann et al., 2006; Granacher et al., 2010a), and an impaired interaction of antagonistic muscles in complex postural tasks (Berger et al., 2010; Hassanlouei et al., 2012; Kennedy et al., 2012), and hence is related to an increased fall incidence (Helbostad et al., 2007; Granacher et al., 2010a). In the majority of protocols, fatigue was induced throughout repetitive contractions of selective muscles (Gandevia, 2001; Paillard, 2012). Although there are a substantial number of articles dealing with the interference of fatigue and body equilibrium, the interpretation is confounded by the variety of paradigms used to study it. Fatigue protocols vary in duration (20 s to 15 min), level of exhaustion (−5 to 70% of maximal voluntary contraction), selected muscle topography (musculature encompassing ankle, knee, hip, torso, or neck), as well as the number of muscles involved in the fatiguing exercise (local vs. whole body fatigue, for review see Paillard, 2012). So far, no study has concentrated on fatigue effects that occur during prolonged balance tasks. Despite its daily life relevance, experiments have not been executed within a realistic fatiguing postural scenario, yet. Thus, for a conclusive statement about the neuromuscular compensation for balance recovery after—or even during—fatigue, further considerations to elucidate neuromuscular mechanisms of postural fatigue are still needed (Paillard, 2012).

With regard to body equilibrium, investigations assessing the effect of fatigue on the ***neuromuscular system*** focused on the redistribution of compensatory muscle activity and resulting reorganization of multi-joint coordination in response to perturbation after exhaustion (Gandevia, 2001; Paillard, 2012). Scientific protocols involve fatiguing repetitions of mono- or poly-segmental movements (i.e., isolated or coupled contractions of tonic muscles, such as the ankle plantar flexors and dorsi flexors or invertors and evertors, knee extensors, hip flexor-extensors, hip abductor-adductors, erector spinae, or neck extensors) or whole body exercise (running, cycling), interfering with postural control (Paillard, 2012). Particular emphasis is put on the reflex arc that governs the operation of immediate muscle contractions, which are of major relevance for a quick readjustment of the body segments to restore equilibrium after perturbation to avoid falls (Granacher et al., 2010a).

Although study findings diverge according to the nature of the protocols, two major conclusions can be drawn. First, the number of muscles stimulated during a fatiguing exercise is related to the magnitude of the postural deterioration (Enoka and Stuart, 1992; Paillard, 2012). Thus, deficits in body equilibrium grow with the number and size of exhausted muscles, ranging from single (focal muscle) to local (mono-articular muscle set) to complex (poly-articular muscle groups) to whole body fatigue (Boyas et al., 2011; Paillard, 2012).

Second, disregarding the protocol and localization of fatigue, muscles of the non-fatigued segments compensate for neuromuscular deficits of the fatigued region. Thus, it can be assumed that fatigue of the proximal musculature (e.g., knee and/or hip muscles) induces the recruitment of distal muscle groups (e.g., ankle muscles) to counteract its disturbing effects on postural control and vice versa (Gribble and Hertel, 2004; Bellew and Fenter, 2006; Salavati et al., 2007; Bizid et al., 2009).

However, there are still questions regarding crucial issues, such as the effect of fatigue on reflex activity, agonist-antagonist muscle coordination and how these aspects are interlinked with neuro-mechanic coupling and balance strategy (Bonnard et al., 1994; Sparto et al., 1997; Herrmann et al., 2006; Berger et al., 2010; Kennedy et al., 2012). For instance, inhomogeneous findings exist for modulations in postural reflex responses. Herrmann et al. (2006) showed a fatigue-induced increase in contrast to Granacher et al. (2010a), who demonstrated a decrease in response to perturbation. In both studies reflex-relevant phase-specific distinctions between postural responses defined as: short (SLR), medium (MLR), and long (LLR) latency reflex responses after perturbation (Horak and Nashner, 1986; Diener et al., 1988; Taube et al., 2006; Rinalduzzi et al., 2015) in addition to H-reflex measures for an assessment of spinal excitability have not yet been implemented (Nielsen and Kagamihara, 1993). The temporal distinction of reflexes, meaning their latency-dependency and ability to modulate on specific levels within the CNS on different pathways, is of high functional relevance (Horak and Nashner, 1986; Diener et al., 1988; Taube et al., 2006; Rinalduzzi et al., 2015). Therefore, it is emphasized that a more comprehensive understanding can be achieved by distinct subdivision of the reflex response, combined with additive methodological approaches, such as peripheral nerve stimulation (PNS, Taube et al., 2006).

Likewise, inconsistencies in antagonistic muscle co-activation in response to fatigue have been reported. On the one hand, some authors have demonstrated an increased co-contraction in the shank muscles (Berger et al., 2010; Granacher et al., 2010a; Kennedy et al., 2012) associated with increased joint stiffening and shifts in balance strategy due to fatigue (Bonnard et al., 1994; Sparto et al., 1997). In contrast, other authors could not confirm these results and demonstrated a decreased co-contraction accompanied by a reduction in joint control (Hassanlouei et al., 2012; Cheng et al., 2015) after fatigue. Although the reasons and consequences for these inconsistencies remain unclear, it can be speculated that contradictions occur due to differences in fatigue protocols that may impact the magnitude and topography of postural impairment (Enoka and Stuart, 1992; Gandevia, 2001).

Despite the widespread relevance of fatigue in different areas of the rehabilitative sports medicine and geriatrics, as well as the substantial number of related articles, the underlying neuromuscular mechanisms in terms of posture control are poorly understood. Therefore, this study aimed to evaluate the effect of ***postural fatigue*** on body equilibrium, neuromuscular control, and joint kinematics in response to perturbation. Fatigue symptoms were deliberately elicited within a balance paradigm to benefit from monitoring the chronological progression of fatigue within the exhausting process, considering both regional and temporal differentiation and affecting the relevant muscle groups. We were particularly interested in identifying if and how fatigue is counterbalanced on a neuromuscular and kinematic level and if this neuro-mechanical coupling can be attributed to particular reflex phases or body segments. We hypothesized that modulations in response to fatigue would be phase (SLR, MLR, and LLR) and segment specific (distal and proximal), and may be associated with differences in the balance strategy, accompanied by differences in kinematic output.

## Materials and methods

### Experimental design

We used a single-group repeated-measures study design to evaluate acute effects of fatigue on postural equilibrium, reflex activity, and balance strategy. For this purpose, two protocols were executed randomly on two different days: the fatigue protocol (FAT), consisting of continuous exposure to perturbations until exhaustion, and the volume-matched control protocol (CON), consisting of consecutive 30 s exposure to eight perturbations which are intermitted by 30 s rest periods (Lesinski et al., 2015). Each i_th_ trial of CON was identical with the i_th_ trial of FAT. For those subjects that performed CON before FAT an upper limit was set for CON. After both protocols have been performed, we deleted the last attempts of CON to individually adjust the number of CON trials to the number of FAT trials. For both protocols, subjects stood on their right leg and perturbations were randomly allocated in eight directions (anterior, posterior, medial, lateral and the four diagonals) in intervals of 4–6 s with an amplitude of 3 cm and a velocity of 1.8 m/s on a Perturmed® (Brüderlin, Göppingen, Germany, Freyler et al., 2015). We chose this experimental setting because fatigue symptoms could be elicited in an all-embracing manner spanned over distal and proximal muscles (disregarding body segment, agonist, or antagonist musculature) and the symptoms could be monitored in continuous progression until exhaustion (Paillard, 2012). The stop criterion was a failure rate of 50% within four perturbations. In order to assess the effects of fatigue on balance control, center of pressure (COP) displacement, joint kinematics, electromyographic (EMG) activity, and spinal excitability at the peak of the short-latency response (SLR) were monitored concomitantly during FAT and CON, after respective posterior perturbation of the foot was carried out. One has to note that this posterior perturbation was executed randomly, but uniformly for both FAT and CON. Prior to data collection, the subjects performed isometric maximal voluntary contractions (MVC) for all recorded muscles (see Freyler et al., 2015 for more details about the procedure of MVC collection).

**Figure 1:**
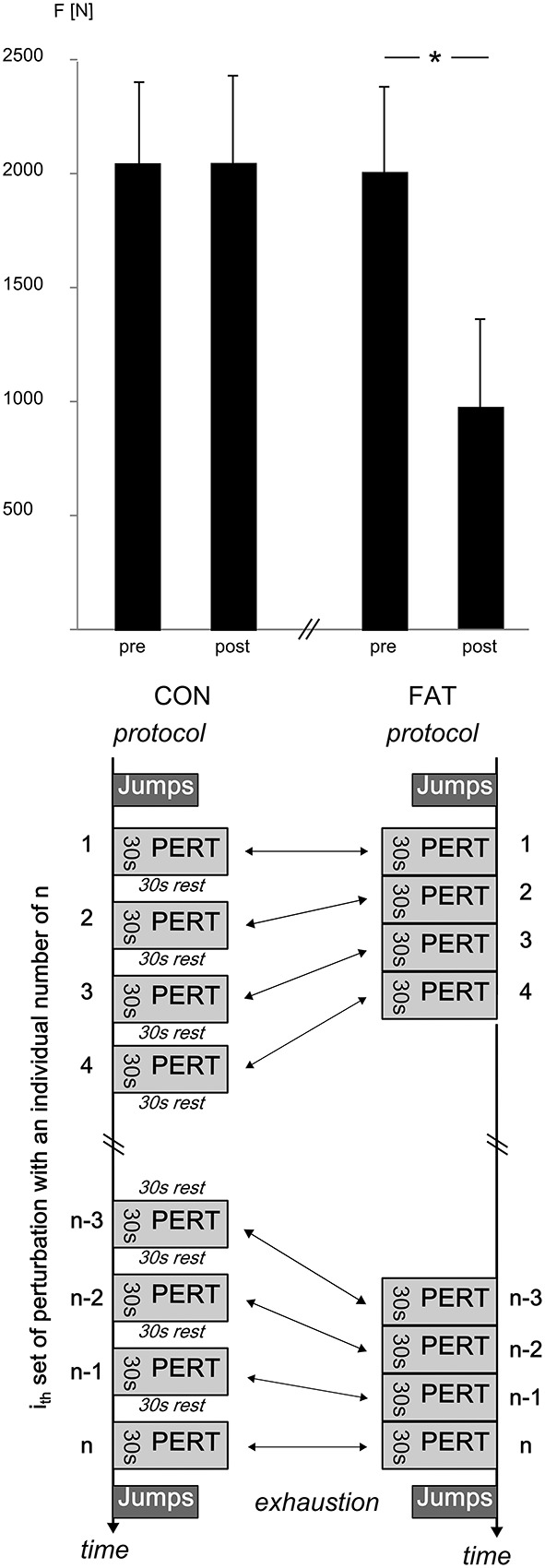
**Top:** Grand means of peak GRFs [F] before and after the CON and FAT protocol. Peak GRF did not change in response to CON; FAT caused a significant reduction in peak GRF. ^*^Displays a significant difference. **Bottom:** CON and FAT protocols with n sets for each protocol, all sets contained eight perturbations in eight different directions with each i_th_ set being equal in order and inter-perturbation breaks for FAT and CON. The n_th_ set was the individually last set for each subject before exhaustion.

Data sets of the FAT and CON protocols were divided into four periods with within-subject equity in time and trial numbers (T_1_, T_2_, T_3_, and T_4_): T_1_ data of the first quarter was averaged, with data for T_2_ and T_3_ of the second and third quarter being averaged and lastly the fourth quarter is presented in T_4_ (Table 1 and Figure 1). The 30 s rest periods for the CON protocol were not relevant for the volume measurements. In this case only the sum of the 30 s perturbation sets were appropriate.

**Table 1:** **Changes in peak GRF and RFD before and after the FAT and CON protocol**.

**Parameter**	**FAT**		**CON**		**rmANOVA**
	**Pre**	**Post**	**Pre**	**Post**	
Peak GRF [N]	2084 ± 401	2109 ± 452	2069 ± 444	989 ± 412	*P* = 0.001 *F* = 7.29
RFD [kN/s]	21 ± 6	13 ± 5	21 ± 6	21 ± 5	*P* = 0.003 *F* = 5.61

*P-and F-values denote rmANOVA time × protocol interaction effects*.

### Subjects

24 subjects (7 females, 17 males, age 25 ± 2 years, height 180 ± 5 cm, weight 74 ± 8 kg; values expressed as mean ± standard deviation) volunteered to participate in this study. All subjects gave written informed consent to the experimental procedure, which was approved by the ethics committee of the University of Freiburg and was in accordance with the latest revision of the Declaration of Helsinki. The subjects were recruited at the sports institute of the University of Freiburg. Thus, we had a homogenous healthy and sportive subpopulation. They were healthy with no previous neurological irregularities or injuries of the lower extremity. A priori, the sample size was estimated by means of a power analysis (*f* = 0.5; alpha = 0.05; power = 0.8).

### Fatigue

As recommended in the literature, strength loss was used as an index of fatigue (Kelly et al., 2005; Paillard, 2012). To monitor fatigue, the subjects performed 10 maximal jumps (both legged hops) before and after FAT and CON on a force platform (Leonardo®, Pforzheim, Deutschland). Peak ground reaction force (GRF) and rate of force development (RFD) was assessed.

### Outcome measures

#### Number of failed attempts

We counted the number of failed attempts. Failed attempts were defined as attempts in which subjects failed to regain postural equilibrium after surface translation and fell (note that due to the safety frame surrounding the subjects, they were secured from falling). Criterions were defined as follows: (i) touching the safety frame of the Perturmed® with at least one hand (Freyler et al., 2015) or (ii) lift off with the unsupported foot to avoid falling. In cases (i) and (ii) subjects would not have been able to regain equilibrium without falling. In contrast, successful attempts were counted when the subjects were able to regain equilibrium within 2 s after surface translation without touching the safety frame. Thus, all attempts in which subjects did not use external support to avoid a fall have been considered to be successful with a regain of equilibrium. Trials were also considered to be successful when subjects: struggled, comprised huge displacements, were swinging, compensated via up or down movements of the COM, demonstrated a fore- or back-wards lean in case they were able to subsequently re-stabilize postural equilibrium.

#### Postural sway

Postural sway was quantified by means of a pressure distribution measuring system (Pedar®, Novel, Germany). The sensor mat was placed on the perturbation platform; the COP was recorded by means of 3D sensor deformation with a 100 Hz sampling rate and a spatial resolution of four sensors per square centimeter. COP displacement (COP_D_) and velocity (COP_V_) were assessed in the anterior-posterior direction and were averaged over the trials for each subject and each of the five conditions (Cabeza-Ruiz et al., 2011).

#### Kinematics

Ankle (dorsiflexion and plantar flexion), knee (flexion and extension) and hip (flexion and extension) joint kinematics in the sagittal plane were recorded using electrogoniometers (Biometrics®, Gwent, UK). Goniometers were fixed at the respective joints according to previous research (Ritzmann et al., 2015). All signals were recorded with a sampling frequency of 1 kHz.

#### EMG recording

Bipolar Ag/AgCl surface electrodes (Ambu Blue Sensor P, Ballerup, Denmark; diameter 9 mm, center-to-center distance 25 mm) were placed over the M. soleus (SOL), medial gastrocnemius (MG), tibialis anterior (TA), rectus femoris (RF), biceps femoris (BF), vastus medialis (VM), and gluteus maximus (GM) muscles of the right leg. The longitudinal axes of the electrodes were in line with the direction of the underlying muscle fibers. The reference electrode was placed on the patella. Interelectrode resistance was kept below 2.5 kΩ by means of shaving, light abrasion, degreasing and disinfection of the skin. The EMG signals were transmitted to the amplifier (band-pass filter 10 Hz to 1 kHz, 1000x amplified) via shielded cables and recorded with 1 kHz. The cables were carefully taped to the skin.

#### H-reflex

Modulation in Ia afferent transmission of the SOL motoneuron pool in response to fatigue was assessed using H-reflex measurements; H-reflexes were elicited via PNS with single rectangular pulses of 1 ms duration (Digitimer DS7, Digitimer, Welwyn Garden City, UK). The cathode (2 cm in diameter) was placed in the popliteal fossa and moved until the best position was found for eliciting an H-reflex in the SOL. The anode (10^*^5 cm dispersal pad) was fixed directly below the patella on the anterior part of the knee. H-reflexes were elicited by electrically stimulating the posterior tibial nerve. Based on previously recorded H/M recruitment curves, stimulation intensities were set to 25% of the maximal M-wave (M_max_) for all measurements (Crone et al., 1987; Taube et al., 2008). PNS was triggered to occur at the peak of the SLR during posterior perturbation.

### Data processing

Concerning the jumps, GRFs were used to determine the peak GRF (push off and landing threshold 3N) and RFD (peak GRF divided by the time from GC until the force signal reached its peak). The mean value of the 10 jumps was used for statistical analysis (see Ritzmann et al., 2011).

Data sets of the FAT and CON protocols were divided into four periods with within-subject equity in time and trial numbers (T_1_, T_2_, T_3_, and T_4_); we executed data processing for these periods as follows:

COP_D_ [mm] was calculated for each perturbation as the difference between the COP peak excursion (defined as the maximum value of the COP excursion within the 400 ms window of perturbation) and the onset position expressed as an absolute value. Only the anterior displacement was considered to be of relevance for data processing, due to the COP shifting contrarily to the perturbation direction i.e., a backward translation of the platform caused a forwards shift of the COP and vice versa (Freyler et al., 2015). COP_V_ was calculated according to Freyler et al. (2015): COP_V_[mm/ms] = *COP*_*D*_/*t* (*t* is defined as the time interval from the onset of perturbation to COP peak excursion).

Ankle, knee, and hip joint kinematics were expressed as joint excursions [°] and calculated as the difference between the peak angle position (defined as the maximum value of the angle excursion within the 400 ms window of perturbation) and the onset position (Freyler et al., 2015) for each perturbation.

For each of the recorded muscles, the EMG signals were rectified and integrated (iEMG [mVs]). For data analysis, iEMG was divided into four relevant phases according to literature before and after perturbation: pre-activation–100–0 ms prior to perturbation (PRE), 30–60 ms (SLR, Rinalduzzi et al., 2015), 60–85 ms (MLR), and 85–120 ms (LLR, Taube et al., 2006) post perturbation. Subsequently, the iEMGs were time normalized [mV/s] and normalized to the MVC [%MVC].

In addition, to assess the simultaneous activation of antagonistic muscles encompassing the ankle, knee and hip joint, the co-contraction index (CCI) was calculated for SOL_TA, VM_BF and GM_RF with the rectified and normalized EMG by means of the following equation: CCI = ∑ CCI_i_, CCI_i_ = ∑ (lower EMG_i_/higher EMG_i_) × (lower EMG_i_+ higher EMG_i_) for each sample point _i_, (Lewek et al., 2004). CCIs were expressed for PRE and the entire reflex phase (RP) 30–120 ms after perturbation.

Peak-to-peak amplitudes of the H-reflexes and M-waves were calculated.

For parameters and subjects, we averaged the values for T_1_, T_2_, T_3_, and T_4._ For EMG, CCI and H-reflexes, we normalized the mean values to T_1_ for the FAT and CON protocols, respectively.

### Statistics

To test for fatigue-induced changes over time, a repeated measures analysis of variance (rmANOVA) was used [time (T_1_, T_2_, T_3_, T_4_) × protocol (FAT vs. CON)]. The normality of the data was evaluated using Kolmogorov-Smirnov test; data followed a normal distribution. If the assumption of sphericity, as measured via Mauchly's sphericity test, was violated, the Greenhouse-Geisser correction was used. *Muscle group* (shank vs. thigh) was included as a within-subject factor to detect differences between the CCIs SOL_TA, VM_BF, and GM_RF. *Phase* was included as a within-subject factor to detect differences between the reflex phases SLR, MLR, and LLR. *Segmentation* (ankle vs. knee vs. hip) was included as a within-subject factor to detect the dependencies of the different joint flexions of the lower extremities. To correct for multiple testing, we used Bonferroni; each *P*-value (*P*_i_) for each test was multiplied by the number of tests (*P*_i adjusted_ = *P*_i_^*^ *n, n* = number of tests). If *P*_i adjusted_ < 0.05, we considered the respective test i to be of statistical significance. A bivariate two-tailed Pearson correlation analysis was executed to determine the strength of linear relations between the number of failed attempts and the iEMG in the reflex phases SLR, MLR, and LLR for all muscles, the CCIs in RP, and the joint excursions. The false discovery rate was controlled according to the Benjamini–Hochberg–Yekutieli method, a less conservative but still stringent statistical approach conceptualizing the rate of type I errors (Benjamini and Hochberg, 1995; Benjamini and Yekutieli, 2005). All analyses were executed using SPSS 20.0 (SPSS, Inc., Chicago, IL, USA). The values are presented as mean values ± standard deviations (M ± SD).

## Results

Changes in peak GRF after FAT and CON are illustrated in Figure 1, grand means for peak GRF and RFD are displayed in Table 1. The rmANOVA revealed a significant *time* × *protocol* interaction effect for RFD and peak GRF, indicating a reduced force generation capacity after FAT. Grand means of the failed attempts are displayed in Table 2.

**Table 2:** **Grand means of the failed attempts, anterior center of pressure (COP) displacement and velocity, and hip, knee and ankle joint kinematics are shown: data are displayed for the fatigue (FAT) and control protocol (CON) for the periods T_**1**_–T_**4**_ (first, second, third, and fourth quarter of the data set including all trial and subjects)**.

**Parameter**		**T_1_**	**T_2_**	**T_3_**	**T_4_**	**rmANOVA**
Failed attempts [%]	FAT^*^ CON	**0.5** ± **0.3** 0.9 ± 0.4	**0.8** ± **0.3** 0.6 ± 0.3	**9.3** ± **12.2** 0.5 ± 0.3	**15.9** ± **13.5** 0.5 ± 0.3	***P*** = **0.001** ***F*** = **10.02**
COP displacement [mm]	FAT^*^ CON	**17.0** ± **10.5** 16.9 ± 10.5	**16.9** ± **10.2** 17.4 ± 11.3	**14.0** ± **8.7** 17.1 ± 12.5	**12.2** ± **8.9** 17.8 ± 12.5	***P*** < **0.001** ***F*** = **9.66**
COP velocity [mm/s]	FAT CON	1.4 ± 0.3 1.3 ± 0.4	1.4 ± 0.2 1.2 ± 0.3	1.5 ± 0.3 1.3 ± 0.5	1.3 ± 0.5 1.4 ± 0.5	*P* = 0.48 *F* = 0.26
Hip joint excursion [°]	FAT^*^ CON	**2.2** ± **1.3** 1.9 ± 1.2	**5.0** ± **1.8** 1.9 ± 1.6	**1.2** ± **0.7** 2.04 ± 1.4	**1.0** ± **0.61** 2.2 ± 1.5	***P*** < **0.001** ***F*** = **47.68**
Knee joint excursion [°]	FAT^*^ CON	**2.9** ± **2.1** 3.1 ± 2.4	**3.7** ± **3.2** 3.0 ± 2.3	**1.6** ± **1.0** 2.7 ± 2.0	**1.2** ± **0.9** 2.7 ± 1.9	***P*** = **0.02** ***F*** = **5.51**
Ankle joint excursion [°]	FAT^*^ CON	**6.2** ± **1.1** 6.3 ± 0.4	**5.5** ± **1.2** 6.2 ± 0.6	**5.3** ± **0.9** 6.2 ± 0.8	**5.1** ± **1.1** 6.0 ± 0.4	***P*** = **0.04** ***F*** = **4.51**

*Data are normalized to baseline values*.

Bold P-and F-values denote significant rmANOVA time x protocol interaction effects, bold letters indicate a significant time effect and are marked with a ^*^.

Note that only successful trials were considered for data analysis.

### Postural sway

Fatigue-induced changes in COP_D_ and COP_V_ are displayed in Table 2. The rmANOVA revealed a significant *time* × *protocol* interaction effect for COP_D_, pointing toward a progressively reduced COP displacement in anterior direction with fatigue.

### Kinematics

**Figure 2:**
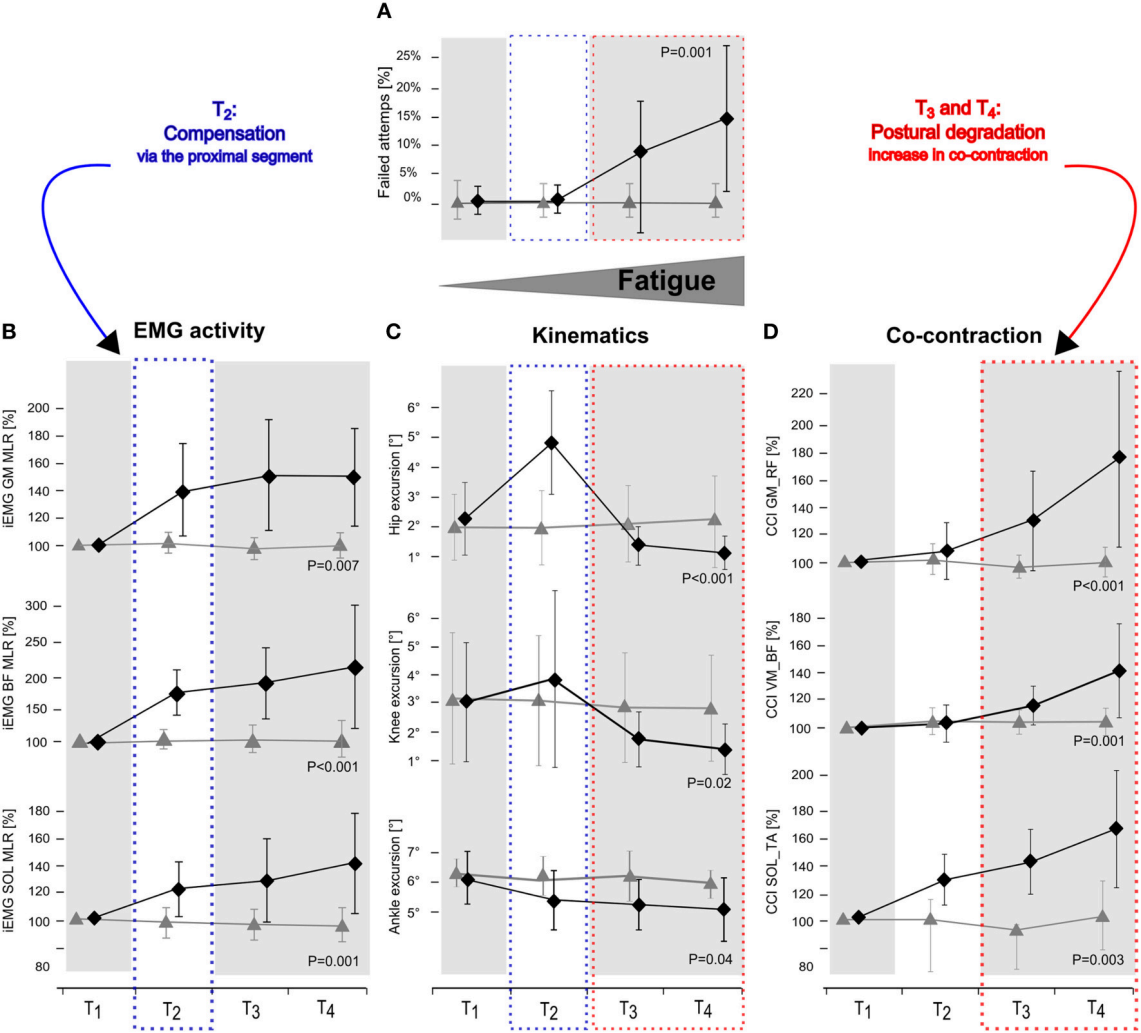
**Changes in number of failed attempts (A), EMG activity in GM, BF, and SOL (B), hip, knee, and ankle joint deflection (C) and Co-contraction index (CCI) in the antagonist muscle groups GM_RF, VM_BF, and SOL_TA (D) in response to posterior surface displacement**. Grand means (♦ fatigue protocol FAT, ▴ control protocol CON) are illustrated for the periods T_1_–T_4_ (first, second, third and fourth quarter: including all trial and subjects). While data showed no changes for CON over time, results reveal segmental compensation for fatigue in T_2_by augmented knee and hip deflections and an elevated level of neuromuscular activation in GM and BF (blue dotted frame) followed by distinctly reduced knee and hip deflections in T_3_ and T_4_ accompanied by increased co-contractions and a raise in number of failed attempts (red dotted frame). *P*-values are given for *time* × *protocol* interaction.

Fatigue-induced changes in joint kinematics are illustrated in Figure 2; grand means are displayed in Table 2. The rmANOVA revealed a significant *time* × *protocol* interaction effect for ankle, knee, and hip joint excursions, indicating a progressive decline in joint excursions with fatigue. Furthermore, the rmANOVA revealed a significant interaction effect for *time* × *protocol* × *segmentation* (*P* = 0.004, *F* = 8.16) indicating segmental dependencies when compensating perturbation along the lower body segment.

### EMG

Fatigue-induced modulations in EMG activity are displayed in Table 3 and Figure 2. The rmANOVA revealed a significant *time* × *protocol* × *phase* interaction effect for GM (*P* = 0.01, *F* = 5.22) and TA (*P* = 0.002, *F* = 11.81). A significant interaction effect was found for *time x protocol x muscle group* for PRE (*P* = 0.007, *F* = 7.19), indicating topographic differences in the muscle pre-setting. Significant phase- and muscle-specific differences were observed for SOL, TA, GM and BF in each of the recorded reflex phases (SLR, MLR, LLR), as well as for RF and VM in MLR and LLR, indicating a progressive and muscle-specific increase in neuromuscular activation in response to fatigue.

**Table 3:** **Changes in neuromuscular activation: Grand means of the iEMGs of GM, RF, VM, BF, SOL, and TA during pre-activation (PRE) and the relevant reflex phases short-, medium-, and long-latency response (SLR, MLR, and LLR)**.

**Parameter**			**T_1_**	**T_2_**	**T_3_**	**T_4_**	**rmANOVA**
iEMG GM [%MVC]	PRE	FAT CON	1.00 ± 0 1.00 ± 0	1.04 ± 0.12 0.99 ± 0.04	1.08 ± 0.19 1.01 ± 0.10	1.12 ± 0.16 1.03 ± 0.10	*P =* 0.12 *F* = 1.49
	SLR	FAT^*^ CON	**1.00** ± **0** 1.00 ± 0	**1.32** ± **0.23** 0.99 ± 0.08	**1.35** ± **0.27** 0.98 ± 0.08	**1.41** ± **0.28** 1.04 ± 0.12	***P*** = **0.02** ***F*** = **3.48**
	MLR	FAT^*^ CON	**1.00** ± **0** 1.00 ± 0	**1.37** ± **0.32** 1.01 ± 0.07	**1.47** ± **0.38** 0.97 ± 0.07	**1.46** ± **0.33** 0.99 ± 0.09	***P*** = **0.007** ***F*** = **7.11**
	LLR	FAT^*^ CON	**1.00** ± **0** 1.00 ± 0	**1.36** ± **0.30** 0.99 ± 0.11	**1.40** ± **0.36** 1.00 ± 0.10	**1.57** ± **0.55** 0.98 ± 0.12	***P*** < **0.001** ***F*** = **15.93**
iEMG RF [%MVC]	PRE	FAT CON	1.00 ± 0 1.00 ± 0	1.05 ± 0.20 0.01 ± 0.16	1.12 ± 0.36 0.99 ± 0.19	1.16 ± 0.41 1.01 ± 0.15	*P* = 0.10 *F* = 1.61
	SLR	FAT^*^ CON	**1.00** ± **0** 1.00 ± 0	**1.00** ± **0.15** 1.00 ± 0.20	**1.34** ± **0.46** 0.98 ± 0.10	**1.48** ± **0.47** 1.00 ± 0.15	*P* = 0.09 *F* = 1.98
	MLR	FAT^*^ CON	**1.00** ± **0** 1.00 ± 0	**1.00** ± **0.13** 0.99 ± 0.13	**1.36** ± **0.37** 0.92 ± 0.17	**1.49** ± **0.40** 0.99 ± 0.21	*P* = 0.06 *F* = 2.31
	LLR	FAT^*^ CON	**1.00** ± **0** 1.00 ± 0	**1.05** ± **0.17** 1.02 ± 0.13	**1.51** ± **0.71** 0.92 ± 0.17	**1.60** ± **0.60** 0.99 ± 0.21	***P*** = **0.006** ***F*** = **16.58**
iEMG VM [%MVC]	PRE	FAT CON	1.00 ± 0 1.00 ± 0	1.04 ± 0.10 0.99 ± 0.17	1.23 ± 0.35 1.00 ± 0.19	1.28 ± 0.51 1.01 ± 0.24	*P* = 0.25 *F* = 0.94
	SLR	FAT CON	1.00 ± 0 1.00 ± 0	1.04 ± 0.14 1.00 ± 0.20	1.49 ± 0.33 1.02 ± 0.19	1.48 ± 0.40 0.99 ± 0.24	*P* = 0.05 *F* = 2.07
	MLR	FAT^*^ CON	**1.00** ± **0** 1.00 ± 0	**1.03** ± **0.17** 1.02 ± 0.22	**1.38** ± **0.46** 1.01 ± 0.16	**1.62** ± **0.64** 1.00 ± 0.22	***P*** < **0.001** ***F*** = **16.77**
	LLR	FAT^*^ CON	**1.00** ± **0** 1.00 ± 0	**1.04** ± **0.11** 1.02 ± 0.12	**1.35** ± **0.30** 1.02 ± 0.18	**1.45** ± **0.52** 1.01 ± 0.21	***P*** = **0.01** ***F*** = **8.10**
iEMG BF [%MVC]	PRE	FAT CON	1.00 ± 0 1.00 ± 0	1.21 ± 0.34 1.03 ± 0.12	1.22 ± 0.31 0.98 ± 0.08	1.25 ± 0.37 1.01 ± 0.14	*P* = 0.11 *F* = 2.55
	SLR	FAT^*^ CON	**1.00** ± **0** 1.00 ± 0	**1.66** ± **0.53** 1.05 ± 0.14	**1.87** ± **0.57** 1.02 ± 0.13	**2.13** ± **0.77** 1.05 ± 0.15	***P*** < **0.001** ***F*** = **2 1.09**
	MLR	FAT^*^ CON	**1.00** ± **0** 1.00 ± 0	**1.73** ± **0.34** 1.03 ± 0.14	**1.86** ± **0.53** 1.04 ± 0.21	**2.11** ± **0.92** 1.04 ± 0.27	***P*** < **0.001** ***F*** = **19.81**
	LLR	FAT^*^ CON	**1.00** ± **0** 1.00 ± 0	**1.67** ± **0.53** 1.02 ± 0.14	**1.90** ± **0.71** 1.06 ± 0.35	**2.07** ± **1.07** 1.00 ± 0.23	***P*** = **0.008** ***F*** = **6.91**
iEMG SOL [%MVC]	PRE	FAT CON	1.00 ± 0 1.00 ± 0	1.16 ± 0.09 0.97 ± 0.06	1.21 ± 0.13 0.97 ± 0.06	1.23 ± 0.19 1.01 ± 0.10	*P* = 0.09 *F* = 2.31
	SLR	FAT^*^ CON	**1.00** ± **0** 1.00 ± 0	**1.12** ± **0.17** 0.99 ± 0.09	**1.23** ± **0.24** 0.97 ± 0.08	**1.33** ± **0.31** 0.97 ± 0.10	***P*** = **0.001** ***F*** = **17.27**
	MLR	FAT^*^ CON	**1.00** ± **0** 1.00 ± 0	**1.26** ± **0.24** 0.97 ± 0.13	**1.34** ± **0.36** 0.95 ± 0.15	**1.49** ± **0.44** 0.95 ± 0.15	***P*** = **0.001** ***F*** = **13.88**
	LLR	FAT^*^ CON	**1.00** ± **0** 1.00 ± 0	**1.24** ± **0.23** 0.98 ± 0.13	**1.32** ± **0.27** 0.97 ± 0.10	**1.46** ± **0.33** 1.95 ± 0.11	***P*** < **0.001** ***F*** = **12.41**
iEMG TA [%MVC]	PRE	FAT^*^ CON	**1.00** ± **0** 1.00 ± 0	**1.34** ± **0.33** 0.90 ± 0.49	**1.42** ± **0.41** 0.91 ± 0.49	**1.57** ± **0.53** 1.02 ± 0.58	***P*** = **0.01** ***F*** = **4.06**
	SLR	FAT^*^ CON	**1.00** ± **0** 1.00 ± 0	**1.42** ± **0.43** 0.94 ± 0.42	**1.50** ± **0.53** 0.92 ± 0.46	**1.53** ± **0.049** 0.96 ± 0.49	***P*** < **0.001** ***F*** = **9.49**
	MLR	FAT^*^ CON	**1.00** ± **0** 1.00 ± 0	**1.41** ± **0 48** 0.99 ± 0.54	**1.35** ± **0.42** 1.01 ± 0.47	**1.47** ± **0.34** 0.96 ± 0.52	***P*** = **0.02** ***F*** = **3.51**
	LLR	FAT^*^ CON	**1.00** ± **0** 1.00 ± 0	**1.38** ± **0.33** 0.84 ± 0.31	**1.40** ± **0.42** 0.86 ± 0.38	**1.34** ± **0.36** 1.00 ± 0.61	***P*** < **0.001** ***F*** = **9.01**

*Data are displayed for the fatigue (FAT) and control protocol (CON) for the periods T_1_–T_4_ (first, second, third, and fourth quarter of the data set including all trial and subjects). Data are normalized to baseline values (T_1_)*.

Bold P-and F-values denote significant rmANOVA time x protocol interaction effects, bold letters indicate a significant time effect and are marked with a ^*^.

### Co-contraction

Fatigue-induced modulations in CCIs are displayed in Table 4 and Figure 2. The rmANOVA revealed a significant *time* × *protocol* × *muscle group* interaction effect for CCI (*P* = 0.009, *F* = 6.31), indicating an overall but segment-specific increase in simultaneously activated agonist muscle groups in response to fatigue. Significant interaction effects *time x protocol* were found for SOL_TA, VM_BF and GM_RF.

**Table 4:** **Changes in neuromuscular activation: Grand means of H-reflex and M-wave amplitudes as well as co-contraction indices (CCI) for the antagonistic muscle groups encompassing the hip joint (GM_RF), knee joint (VM_BF), and ankle joint (SOL_TA) during pre-activation (PRE) and the entire reflex phase (RP, 30–120ms post perturbation)**.

**Parameter**			**T_1_**	**T_2_**	**T_3_**	**T_4_**	**rmANOVA**
H-reflex SOL [%]		FAT^*^ CON	**1.00** ± **0** 1.00 ± 0	**0.97** ± **0.22** 1.03 ± 0.18	**0.92** ± **0.17** 1.00 ± 0.24	**0.86** ± **0.18** 0.99 ± 0.22	***P*** = **0.009** ***F*** = **6.40**
M-wave SOL [%]		FAT CON	1.00 ± 0 1.00 ± 0	1.03 ± 0.20 1.01 ± 0.19	0.94 ± 0.30 0.99 ± 0.24	0.99 ± 0.24 1.01 ± 0.28	*P* = 0.98 *F* = 0.06
CCI GM_RF	PRE	FAT^*^ CON	**1.00** ± **0** 1.00 ± 0	**1.07** ± **0.25** 0.99 ± 0.16	**1.11** ± **0.62** 0.96 ± 0.08	**1.11** ± **0.38** 0.99 ± 0.12	*P* = 0.69 *F* = 0.49
	RP	FAT^*^ CON	**1.00** ± **0** 1.00 ± 0	**1.08** ± **0.21** 1.02 ± 0.11	**1.30** ± **0.37** 0.97 ± 0.08	**1.76** ± **0.65** 1.00 ± 0.11	***P***<**0.001** ***F*** = **11.92**
CCI VM_BF	PRE	FAT CON	1.00 ± 0 1.00 ± 0	1.01 ± 0.20 1.04 ± 0.09	1.15 ± 0.31 1.02 ± 0.09	1.15 ± 0.36 1.02 ± 0.17	*P* = 0.82 *F* = 0.30
	RP	FAT^*^ CON	**1.00** ± **0** **1.00** ± **0**	**1.03** ± **0.13** **1.05** ± **0.10**	**1.16** ± **0.14** **1.04** ± **0.09**	**1.40** ± **0.33** **1.04** ± **0.10**	***P*** = **0.001** ***F*** = **9.72**
CCI SOL_TA	PRE	FAT CON	1.00 ± 0 1.00 ± 0	1.12 ± 0.34 1.04 ± 0.51	1.17 ± 0.48 0.94 ± 0.53	1.15 ± 0.21 0.91 ± 0.39	*P* = 0.78 *F* = 0.36
	RP	FAT^*^ CON	**1.00** ± **0** 1.00 ± 0	**1.28** ± **0.19** 1.00 ± 0.27	**1.41** ± **0.24** 0.91 ± 0.16	**1.64** ± **0.43** 1.02 ± 0.25	***P*** = **0.003** ***F*** = **5.87**

*Data are displayed for the fatigue (FAT) and control protocol (CON) for the periods T_1_–T_4_ (first, second, third, and fourth quarter of the data set including all trial and subjects). Data are normalized to baseline values*.

Bold P-and F-values denote significant rmANOVA time x protocol interaction effects, bold letters indicate a significant time effect and are marked with a ^*^.

### H-reflex

Fatigue-induced modulations in H-Reflex and M-wave amplitudes are displayed in Table 4. The rmANOVA revealed a significant *time x protocol* interaction effect for the H-reflex amplitude, indicating a fatigue-induced reduction in spinal excitability. M-wave amplitudes and SOL activity during PRE remained unchanged.

### Correlations

We detected a significant positive correlation between the number of failed attempts in response to perturbation and CCI of VM_BF (*r* = 0.49; *P* = 0.02) and GM_RF (*r* = 0.72; *P* = 0.009), indicating that an increased fall incidence is associated with higher antagonistic co-contraction in the proximal limb segment. Furthermore, angular hip excursion was negatively correlated to the number of failed attempts (*r* = −0.60; *P* = 0.01). Dependency analysis considering iEMG variables remained insignificant.

## Discussion

The objective of this study was to ascertain the effect of postural fatigue on body equilibrium and to compile knowledge about its influence on neuromuscular control and joint kinematics. The study revealed four major findings: Under exhaustion (i) the number of failed attempts in response to perturbation increased while COP amplitude, as well as angular excursions, decreased. These kinematic changes were accompanied by (ii) an increasing co-contraction in the antagonistic muscles encompassing the limb joints and a declined spinal excitability in SOL. (iii) The number of failed attempts positively correlated with antagonistic co-contraction of the upper leg muscles. (iv) All adaptations were progressively augmented with increasing exhaustion and occurred in the distal, prior to proximal, segment.

In contrast to previously published articles in which fatigue was locally induced in particular muscles groups and effects were assessed before and after the protocol (Madigan et al., 2006; Wilson et al., 2006; Kanekar et al., 2008; Berger et al., 2010; Granacher et al., 2010a,b; Bisson et al., 2011; Boyas et al., 2011), in our study, fatigue symptoms were elicited within the balance paradigm in a holistic manner, integrating all body segments. Closer to daily life scenarios when postural equilibrium is deteriorated throughout repetitive balancing, this study gives particular insight into the neuro-mechanical details. This approach is innovative as it allows for monitoring chronological progression of fatigue within the exhausting process, considering both regional and temporal differentiation. Exhaustion was controlled using force measures, demonstrating a loss in *F*_max_ that exceeds 50%, which is sufficiently high for inducing a deterioration of postural control (Kelly et al., 2005; Paillard, 2012). The experiment has been performed in a specific subpopulation of healthy and sportive students with a high-leveled and diversified training. A conclusive statement for other samples such as for geriatric or pathological patients, as well as sedentary, old, adolescent, or rehabilitated subjects cannot be given, as they may respond differently to fatigue.

### Fatigue mechanisms

The fatigue-induced loss in balance performance is associated with a reduced capability to compensate for external perturbation and is reflected by an increased number of failed attempts, referring to fatigue-induced modulations within the neuro-mechanical coupling: a progressively augmented muscle co-contraction caused by simultaneously activated antagonistic muscles encompassing the ankle, knee, and hip joint was observed, concomitant with a rigid articular stiffening of posture. The increased co-contractions may be ascribed to increased motoneuron discharge frequencies and a successive recruitment of motor units (Enoka and Stuart, 1992; Gandevia, 2001), most likely having a substantial impact on spinal excitability: the gradual reduction in H-reflex amplitude is supposed to be attributed to reciprocal inhibition due to an augmented level of activation in the antagonists (Nielsen and Kagamihara, 1993). Reciprocal inhibition is defined as the antagonist alpha motor neuron inhibition, which is evoked by contraction of the agonist muscle (Crone et al., 1987). As TA activity increases with fatigue-induced exhaustion (Table 2), SOL motoneuron excitability may be inhibited and Ia afferent transmission reduced.

Assessing posture in light of neuromuscular aspects, it is known from the literature that subjects with a greater antagonistic co-contraction of leg muscles, coupled with diminished articular deflections, and COP displacements in response to perturbation, display an augmented fall incidence compared to subjects with a smaller co-contraction (Bruhn et al., 2004; Hortobágyi et al., 2009; Nagai et al., 2011; Sayenko et al., 2012). This may be associated with the number of failed attempts, which although rejected for data analysis, may be associated with the presetting of co-contracted musculature (Bruhn et al., 2004; Hortobágyi et al., 2009; Nagai et al., 2011; Sayenko et al., 2012). We, therefore, argue that such fatigue-induced rigid joint stiffening could compromise the ability to react precisely to sudden surface displacements and to move the center of mass accurately above the base of support (Figure 2), and hence could explain the decline in balance performance in T_3_ and T_4_ in our study (Allum et al., 2002; Tucker et al., 2008).

As only successful trials were analyzed and failed attempts were excluded from data processing, although failures increased over time in FAT, one could argue that this may cause confounding effects. Particularly, as the number of trials in FAT and CON differed by the number of failed attempts. Failed attempts were defined as falling attempts without a regain of balance. They were not considered for data analysis because we were interested in how our healthy sample compensates for fatigue symptoms without losing balance. Thus, we wanted to know which maneuvers and strategies dominate, when postural safety is required although subjects are impaired due to fatigue-induced modulations reducing the normal motor repertoire.

### Fatigue compensation: absorption in the upper leg segment

Our results furthermore indicate that postural fatigue was compensated for in T_2_: The number of failed attempts remained unchanged in T_2_, although fatigue-induced effects in EMG and kinematics within the lower limb segment were evident (Figure 2). It is supposed that in particular the increased EMG activity of BF and GM in T_2_, concomitant with distinctly augmented joint deflections in the knee and hip joints, indicating a shift in activation topography from distal (e.g., ankle muscles) to proximal (e.g., knee and/or hip muscles) muscles that absorbed distal deficits and helped to counteract perturbations to keep the number of failed attempts low as indicated by the interaction effect of *segmentation*. In conjunction with Wilson et al. (2006), who ascertained a general fatigue-induced shift toward the hip strategy, it can be emphasized that distal impairments can be absorbed at the proximal region (Figure 2). In Concerning the underlying mechanisms, activation intensities were particularly high in MLR and LLR. Both reflex phases are supposed to rely on polysynaptic pathways with functional significance to induce appropriate active joint moments for the preservation of postural stability (Nashner, 1977; Horak and Nashner, 1986; Dietz et al., 1988, 1989). Thus, our findings indicate that a reacquisition of center of mass stabilization relies on using long loop reflexes, with the goal to control trunk movements in order to achieve a fast balance recovery in response to surface translation, absorbs fatigue-induced distal deficits (Szturm and Fallang, 1998).

### Fatigue impact: segmental distinction

Failed attempts started to considerably appear in T_3_ and were progressively augmented with increasing exhaustion in T_4_. Correlations additionally indicate that an increased fall incidence interrelates with higher upper limb, but not lower limb, co-contraction and rigidity, or any other change in neuromuscular activation. Hence, based on our results, we assume that monopedal equilibrium is less disturbed as a result of fatigue in the distal compared to the proximal musculature. This observation is in accordance with previous literature regarding balance research. There is evidence that fatigue symptoms elicited in hip or knee musculature affect postural control, while fatigue of muscles encompassing the ankle joint has less or the same impact on body equilibrium (Miller and Bird, 1976; Gribble and Hertel, 2004; Salavati et al., 2007; Bizid et al., 2009; Bisson et al., 2011). This is also in line with the interaction effect of *segmentation:* As the lower extremities' segments are interconnected, deficits in neuromuscular activation may cause dependencies reflected in joint deflections (Freyler et al., 2015). Thus, our findings reveal that postural fatigue is a length-dependent phenomenon occurring in the distal prior to the proximal musculature. We observed a reorganization of multi-joint coordination, recruiting proximal muscles, and using the upper limb segment to efficiently compensate for distal deficits.

In ***conclusion***, outcomes address functional relevance and practical application of postural fatigue, identifying two major characteristics within chronological progression of exhaustion in healthy and active subjects: At first, fatigue causes a redistribution of active muscles and a reorganization of multi-joint coordination to stabilize equilibrium. As postural fatigue occurred as a length-dependent phenomenon appearing in the distal prior to the proximal musculature, a priori, the neuromuscular system takes advantage of a shift from the distal to proximal segment for controlling posture and fall avoidance. Second, in contrast to proximal deficits, distal fatigue was not neglectable and considerably affected postural safety and increased fall incidence. For postural safety, in conjunction with fatigue, muscles encompassing the hip, and knee joint seem to be of superior importance.

It may be speculated that elderly, geriatric, and adolescent subjects or patient groups may respond differently to fatigue compared to our healthy and well-trained sample. As a prospective for further research focusing on the interrelation of fatigue and posture control, investigations in a wider spectrum of subjects presenting different subpopulations would be of particular interest.

## Author contributions

All authors RR, KF, AW, and AG made substantial contributions to the conception or design of the work, the acquisition, analysis, and interpretation of data for the work. Further they contributed drafting the work and revising it critically, they helped with the final approval of the version to be published and made the agreement to be accountable for all aspects of the work in ensuring that questions related to the accuracy or integrity of any part of the work are appropriately investigated and resolved.

### Conflict of interest statement

The authors declare that the research was conducted in the absence of any commercial or financial relationships that could be construed as a potential conflict of interest. The Review Editor TK and handling Editor JS declared their shared affiliation, and the handling Editor states that the process nevertheless met the standards of a fair and objective review.
